# Spatial–temporal variation and source analysis of heavy metals in different land use types in Beilun District (2015 and 2022)

**DOI:** 10.1038/s41598-024-65811-w

**Published:** 2024-07-02

**Authors:** Pengwei Zhang, Lanfang Hu, Bo Gao, Feng Gao, Xuchu Zhu, Yaying Li, Huaiying Yao

**Affiliations:** 1 Key Laboratory of Urban Environment and Health, Institute of Urban Environment, Chinese Academy of Sciences, Xiamen, 361021 China; 2 Zhejiang Key Laboratory of Urban Environmental Processes and Pollution Control, Ningbo (Beilun), Zhongke Haixi Industry Technology Innovation Center, Ningbo, 315800 China; 3 College of Environmental Science and Engineering, Guilin University of Technology, Guilin, 541004 China; 4 School of Environmental Ecology and Biological Engineering, Research Center for Environmental Ecology and Engineering, Wuhan Institute of Technology, Wuhan, 430205 China; 5 Beilun Ningbo Environmental Protection Agency, Ningbo, 315800 China

**Keywords:** Heavy metals, Urban and peri-urban soil, Spatial–temporal distribution, Pollution index, Source analysis, Environmental sciences, Environmental impact

## Abstract

The soil environment plays an important role in urban ecosystems. To study the heavy metal contamination of soil in Beilun District, Ningbo, we collected soil samples from 60 points in urban and peri-urban areas of Beilun District and analyzed the spatiotemporal variation and sources of heavy metal pollution in various land-use types. The results shown that the heavy metal contents in 2015 and 2022 were higher than the background soil values of Ningbo city, and there was an accumulation of heavy metals over these 7 years. The contents of heavy metals in green belts and woodland in 2022 were higher than those in 2015, while there was no significant change in agricultural land. The heavy metal contents in both years were mainly in the order green belts > agricultural land > woodland. The spatiotemporal distribution of heavy metal content showed that heavy metal pollution in Beilun District was concentrated in five industrial areas, and there was a trend toward the disappearance of highly polluted points. But the single-factor pollution index, pollution load index (PLI), and geoaccumulation index (I_geo_) indicated that there was no significant heavy metal pollution in Beilun District, and individual elements at specific points showed slight pollution. The source analysis results showed that the main source of Hg is chemical, As is mainly derived from agricultural, Cr, Ni and Cu are mainly derived from natural, the main sources of Zn and Cd are electroplating and machinery activities, and the main source of Pb is traffic. These results specify a reference for future investigation on urban soil heavy metals, and the source apportionment results provide a scientific foundation for subsequent soil heavy metal pollution treatment.

## Introduction

As a pivot point for material exchange and energy interchanges among the atmosphere, hydrosphere, lithosphere and biosphere, the pedosphere is one of most important reserves and sinks for heavy metals and other pollutants^1–4^. Urban soil is the core of the urban environment. Rapid modern urbanization and population growth impose heavy burdens on urban soil through the input of various pollutants^4,5^. Heavy metals have attracted widespread attention due to their toxicity, bioaccumulation and persistence, which decrease the environmental soil quality and atmosphere and jeopardize human and animal health through the food cycle and dust inhalation^2,6–8^. Some scholars have investigated the concentrations of soil heavy metals in national forest parks^9^, agricultural lands^10^, peri-urban areas^11^ and urban areas^12^. The degree of anthropogenic heavy metal pollution in soil differs from national forest parks to urban areas, and the more economically developed the study area is, the more severe the pollution. Several studies reported the accumulation of heavy metals in different regions and divided the heavy metal sources into two categories: natural geological sources and human activities^13–15^. Natural geological sources mainly comprise soil parent material, rock weathering, forest fires and oceans^16,17^. The main human activity sources are mining, smelting, pesticides, insecticides, industrial waste, transportation and energy production^18^. Multivariate statistical model including positive matrix factorization model (PMF) and principal component analysis-multiple linear regression model (PCA-MLR) are widely used to distinguish between natural and anthropogenic of heavy metal sources^19^. Quantitative source analysis methods are highly important for heavy metal pollution control^20^.

Peri-urban areas are the transition zones between urban and rural areas^21^. These are critical areas that are necessary for urban development and provide materials for urban daily life and production^22^. The accumulation of heavy metals in peri-urban soils has high spatial variability and is affected by land-use type, surface structure, population and climatic conditions^23^. Many studies have found significant differences in heavy metal contents among diverse land-use types^24,25^. For example, Praveena et al. found that land-use type is the main contributor to heavy metal exposure, and the soil heavy metal content in areas adjacent to roads was much higher than that in light industrial areas^26^. Some scholars have also found that the distribution of soil heavy metals in China is ranked as follows: mines > industrial areas > commercial areas > agricultural land > woodland^24,27–29^.

The spatial distribution characteristics and spatiotemporal variability of heavy metals can be intuitively analyzed with multivariate statistical methods and geostatistical analysis methods^30,31^. Nickel et al. explored the effects of land-use type and population on the distributions of Hg, Cd and Pb via multiple regression kriging and generalized linear models and reported that the accumulation of soil Pb decreased from 1995 to 2010^32^. Shi et al. analyzed the spatiotemporal distribution characteristics of soil heavy metals across China, as well as their ecological and human health risks^28^. Yang et al. and Li et al. analyzed the spatial distribution and change trend of heavy metals in Wuhan city and Changxing County by using the kriging spatial interpolation method^33,34^. The multivariate geostatistical method is also an effective way to differentiate the characteristics of soil heavy metal contamination and identify its sources^35,36^. There is no systematic and complete source analysis method. The commonly used source analysis methods include absolute PCA-multiple linear regression, PMF, and the UNMIX model^37^. These methods, which can quantitatively analyze pollution sources, allocate risk based on the contributions of pollution sources and effectively identify pollution sources by combining geostatistical methods and land use data, are highly important for pollution control and management^38,39^. For example, Ninos et al. used kriging space interpolation and PCA to identify the pollution sources of seven heavy metals in soil^40^. Long et al. identified the heavy metal sources in soil and dust in Panzhihua city via analysis of land-use type and PCA^41^. Zhang et al. identified heavy metal sources in soil in the sediments of Dongping Lake by spatial distribution and PMF^42^.

Ningbo is a well-known coastal city in China, and Beilun District is the economic innovation center with the richest port resources in Ningbo. There are five national economic development zones in Beilun District, with industrial parks engaged in activities such as machinery manufacturing, thermal power generation, and petrochemical production. However, rapid economic development has also brought safety hazards such as heavy metal pollution. In this study, the spatiotemporal variability of heavy metals in Beilun District was analyzed based on the spatial distribution characteristics of heavy metals and land use type, and the sources of heavy metals were analyzed by using PMF and absolute PCA-multiple linear regression (MLR). The aims of this study were to (1) analyze the pollution situation of heavy metals in urban and the differences among different land-use types; (2) analyze the spatiotemporal variability of heavy metals in Beilun District from 2015 to 2022 and explore the reasons for the observed trends; and (3) analyze the heavy metal sources in Beilun District by assessing both spatial distribution and land-use type. The results of this study verify the feasibility of spatial distribution and source analysis methods and provide an important reference for the control and treatment of heavy metal pollution in Beilun District.

## Materials and methods

### Study area description and sampling

**Figure 1:**
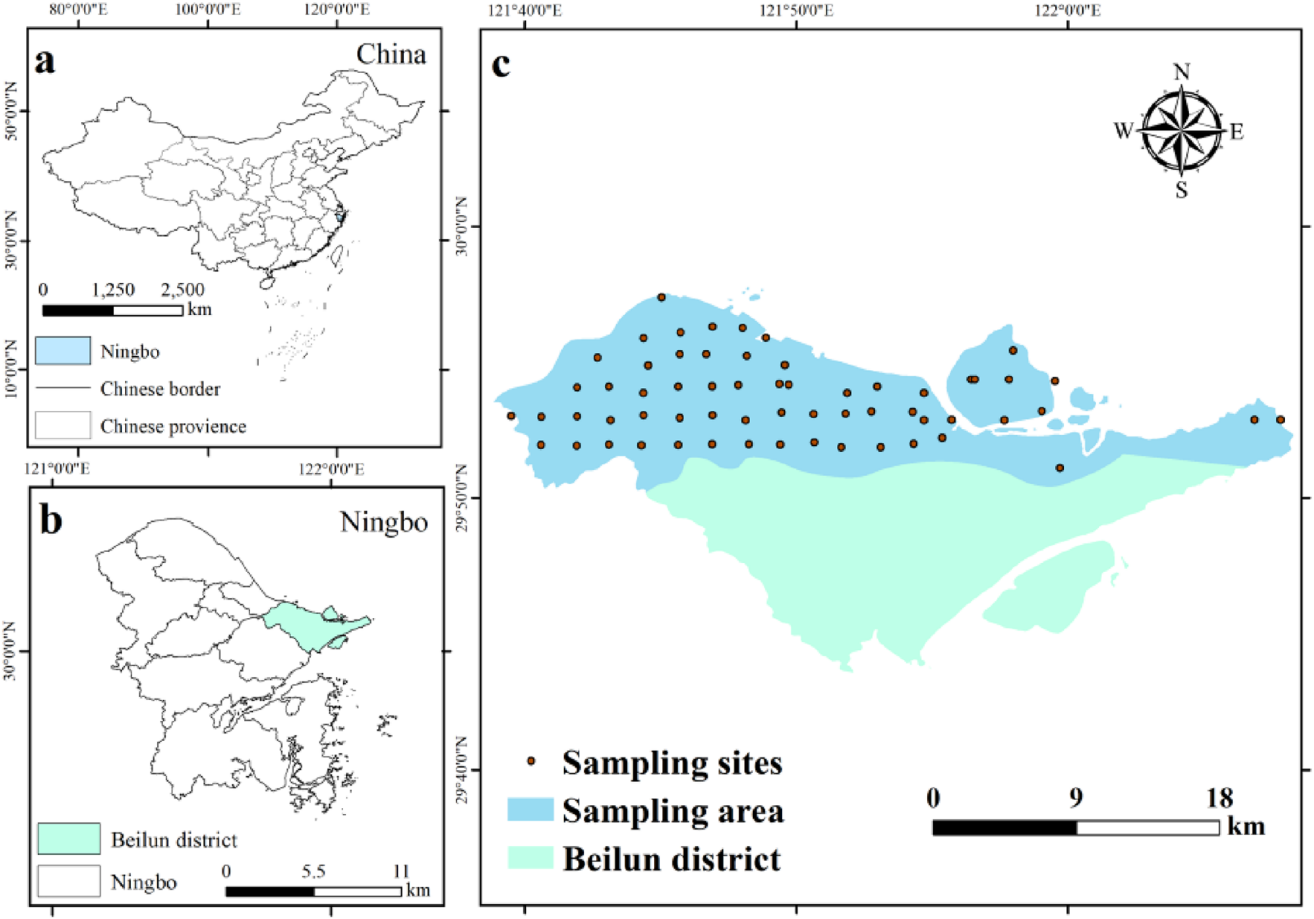
Schematic diagram of heavy metal sampling points in Beilun District (Location of Ningbo in China (**a**), Location of Beilun District in Ningbo (**b**) and Sampling Site Location Distribution (**c**)).

Ningbo is located on the eastern coast of China. Beilun District is located in the northeastern part of Ningbo, with geographical coordinates of 121° 27′ 40″ to 122° 10′ 22″ east longitude and from 29° 41′ 44″ to 29° 58′ 48″ north latitude. As of 2021, the total population of the district was 444,300. Beilun District has five national-level economic development zones, six major industries near the port, and six growing industries, with sustained and rapid economic development. However, the rapid development and industrial activities may also impose a certain degree of pressure on the quality of the regional soil environment. Therefore, grid sampling was performed in Beilun District to study the soil heavy metal pollution. The urban portion of Beilun District is mainly concentrated in the north, and the southern part is mostly suburban and mountainous. In this study, we collected samples from 60 sites in northern urban and peri-urban soils based on the industry-dominant situation in Beilun District. The locations of the sample points are shown in Fig. 1. The agricultural land is primarily used for planting rice (60%) and ornamental plants (40%), with nitrogen, phosphorus, and potassium fertilizers being primarily applied. The annual fertilizer application rate for agricultural land is 1.5–2 kg/ha.

### Sampling and analysis

A soil drill was used to collect the topsoil layer to a depth of 20 cm. Samples collected on the same day were brought back to the laboratory for air drying, and all soil samples were screened through 10 mesh for soil pH determination. A 100-mesh sieve was used to separate soil for heavy metal content analysis. A total of 0.2 g of sample (accurate to 0.0010 g) was weighed using a balance and placed in a digestion tube (a long-handled utensil was used to place the sample at the bottom of the digestion tube). Two drops of ultrapure water were added to moisten the soil. To the digestion tube, add 6 ml of nitric acid, 3 ml of hydrochloric acid, and 2 ml of hydrofluoric acid, all of which are guaranteed reagents. The lid was closed, and the digestion tube was placed on a rotator for digestion in a microwave digester. Before utilizing the microwave digester, activate the magnetron fan in the “Tools” menu under “Diagnostics” to run for 15 min, then turn it off. Follow the temperature ramping procedure of 120 °C, then 160 °C, and finally 190 °C for the digestion. After digestion, the lid was unscrewed once the internal and external atmospheric pressures were consistent. The digestion tube was placed in the device and heated at 140 °C until the volume of solution in the tube reached 1–2 ml. Then, 5 ml of nitric acid was added, and digestion was continued until the volume again reached 1–2 ml (at this point, the solution was viscous). The solution inside the digestion tube was transferred to a 50 ml colorimetric tube with ultrapure water, and ultrapure water was set the volume to the scale mark. The lysate in the colorimetric tube was filtered into a 50 ml transparent plastic bottle with a 0.45 μl filter membrane, and the heavy metal content was determined by inductively coupled plasma‒mass spectrometry (ICP-MS, Thermo Fisher ICAP Q, Waltham, MA, USA)^43^. During the determination process, standard soil samples, parallel samples, and blank samples (SiO_2_) were tested for quality control. In the determination process, quality control tests were conducted on standard soil samples GBW07416a (ASA-5a), GBW07454 (GSS-25), and blank samples (silica dioxide). The recovery rates of heavy metals were 61.3–91.1% (ASA-5a) and 81.6–146% (GSS-25), respectively.

### Pollution evaluation

The single-factor pollution index ${P}_{i}$ uses measured data and soil standard values to simply evaluate the pollution degree of a pollutant^44^. Its formula is shown in Eq. (1).

1 $${P}_{i}=\frac{{C}_{i}}{{S}_{i}},$$

where ${C}_{i}$ signifies the concentration of pollutant i and ${S}_{i}$ signifies the standard value for contaminant i.

The standard values in this study were taken from the “Soil environmental quality risk control standard for soil pollution of agricultural land” (GB15618-2018) and “Soil environmental quality risk control standard for soil pollution of development land” (GB36600-2018).

The pollution load index (PLI) is a parameter used to quantitative metric for evaluating the comprehensive pollution degree of multiple heavy metals in a study area and can intuitively display the contribution of each heavy metal to the pollution and the spatiotemporal trend of heavy metal changes^45^. The uniqueness of this method lies in its high degree of generalization, making it very suitable for the comparison of pollution in multiple regions by calculating the regional index from point index values, and its formula is shown in Eq. (2).

2 $${A}_{i}=\frac{{C}_{i}}{{S}_{i}},$$

where ${S}_{i}$ signifies the background value of contaminant i. Here, the background values of soil elements in Ningbo were used^46^.

3 $$PLI=\sqrt[n]{{A}_{1}\times {A}_{2}\times {A}_{3}\cdots {A}_{n}}$$

where ${A}_{n}$ signifies the pollution coefficient of heavy metal i and n is the number of different heavy metal elements evaluated.

The PLI of an area can be calculated as Eq. (4).

4 $${PLI}_{zone}=\sqrt[n]{{PLI}_{1}\times {PLI}_{2}\times {PLI}_{3}\cdots {PLI}_{n}},$$

where ${PLI}_{n}$ signifies the pollution load index of a given point and n signifies the number of points evaluated. PLI values indicate the level of pollution, with PLI ≤ 1 indicating no pollution, 1 < PLI ≤ 2 indicating a light pollution load, 2 < PLI ≤ 3 indicating a moderate pollution load, and PLI > 3 indicating a strong pollution load.

The geoaccumulation index (I_geo_) was first proposed by the German scholar Muller in 1969 and can quantitatively reflect the level of soil heavy metal pollution. This classification method considers the influence of both human activities and natural conditions on the parent material. It comprehensively evaluate the impact of natural geological activities on the background value and the effect of human activities on the heavy metal content. This approach closely reflects the heavy metal pollution caused by human activities^47^, and its formula is shown in Eq. (5).

5 $${I}_{geo}={\text{log}}_{2}\left(\frac{{C}_{i}}{k\times {S}_{i}}\right),$$

where k is the correction coefficient, which is generally taken as 1.5. The classification criteria are as Table 1.

**Table 1:** Grading criteria for the geo-accumulation index.

I_geo_	Grades
I_geo_ < 0	Unpolluted
0 ≤ I_geo_ < 1	Unpolluted to moderately polluted
1 ≤ I_geo_ < 2	Moderately polluted
2 ≤ I_geo_ < 3	Moderately to heavily polluted
3 ≤ I_geo_ < 4	Heavily polluted
4 ≤ I_geo_ < 5	Heavily to extremely polluted
I_geo_ ≥ 5	Extremely polluted

### Pollution source analysis

PMF is a multivariate factor analysis method that does not require unnecessary measurements of source components. It has the unique ability to impose nonnegative constraints, optimizes the results using the standard deviation of the data, and can produce results even with missing and inaccurate data^48^. Its calculation formula is shown in Eq. (6).

6 $${X}_{ij}=\sum_{k=1}^{p}{G}_{ik}{F}_{jk}+{E}_{ij},$$

where p signifies the number of pollution sources; ${X}_{ij}$ signifies the mass concentration of the jth element in sample i; ${G}_{ik}$ signifies the contribution of source k to sample i; ${F}_{jk}$ signifies the mass concentration of the jth element in source k; and ${E}_{ij}$ signifies the residual matrix.

The equation for calculating the objective function Q is shown in Eq. (7).

7 $$Q=\sum_{i=1}^{n}\times \sum_{i=1}^{m}{\left(\frac{{E}_{ij}}{{U}_{ij}}\right)}^{2},$$

where m signifies the number of elements and ${U}_{ij}$ signifies the uncertainty of the jth element in the ith sample. When the concentration of an element is below the method detection limit (MDL), the uncertainty is 5/6 of the MDL. When the concentration of the element exceeds the MDL, the uncertainty is calculated as Eq. (8).

8 $${U}_{ij}=\sqrt{{\left(\delta \times {C}_{i}\right)}^{2}+{\left(0.5MDL\right)}^{2}},$$

where $\delta$ signifies the relative deviation, which is generally set to 5%. The sources of heavy metals in Beilun District were analyzed using EPA PMF5.0. The number of factors was varied between 3 and 7, and 20 iterations were performed. The data were the most stable when the number of factors was 5. The coefficient of variation (r^2^) between the observed and predicted values of the eight heavy metals ranged between 0.6222 (Cu) and 0.9993 (As). The residuals were mostly in the − 3 to 3 range, and the Q_Robust_ and Q_Ture_ values were close and stable. This indicates that the PMF model fit was good.

PCA is often combined with geostatistical analysis to identify the source of soil heavy metal pollution^14^, and the absolute principal component score (APCS) obtained on this basis can also be used in conjunction with MLR equations to quantify the absolute contribution of each origin of heavy metal pollutions^49^. The analysis steps are as follows.

The data must be analyzed via the Kaiser‒Meyer‒Olkin (KMO) and Bartley tests before PCA is performed. PCA can be performed when KMO > 0.5 and the significance of the Bartley test < 0.05. The KMO values of the data in this study were 0.717 > 0.5, and the significance of the Bartlett test was < 0.05, which indicates that the data in this paper conform to the normal distribution and are suitable for PCA. To determine the source and contribution rate of individual heavy metals, the score of each principal component was used to calculate the APCS. The contribution rate of individual heavy metals was calculated by MLR. The study utilized APCS as the independent variable and the eight heavy metals as the dependent variables.

The first step is to standardize the content of each metal. Its calculation formula is shown in Eq. (9).

9 $${Z}_{ij}=\frac{{C}_{ij}-\overline{{C }_{i}}}{{\delta }_{i}},$$

where ${Z}_{ij}$ signifies a normalized value; ${C}_{ij}$ signifies the measured content of heavy metal i; $\overline{{C }_{i}}$ signifies the average content of heavy metals; and ${\delta }_{i}$ signifies the standard deviation of heavy metal contents.

The composition matrix is obtained by normalizing the data after dimensionality reduction. In the second step, an artificial sample with a content of zero is inserted, and APCS is calculated by subtracting the score of the artificial sample from the principal component score. Finally, the contribution rate of each heavy metal is calculated using MLR. The formula is shown in Eqs. (10) to (12).

10 $${\left({Z}_{0}\right)}_{i}=\frac{0-\overline{{C }_{i}}}{{\delta }_{i}}=-\frac{\overline{{C }_{i}}}{{\delta }_{i}},$$

11 $${C}_{i}={\left({b}_{0}\right)}_{i}+{\sum }_{p=1}^{k}{b}_{pi}\times {APCS}_{p},$$

12 $${RC}_{pi}=\frac{\left|{b}_{pi}\times \overline{{APCS }_{p}}\right|}{{\sum }_{p=1}^{k}\left|{b}_{pi}\times \overline{{APCS }_{p}}\right|},$$

where ${b}_{pi}$ signifies the regression coefficient of factor p for heavy metal i; ${\left({b}_{0}\right)}_{i}$ signifies the constant term of a multivariate linear equation; ${b}_{pi}\times {APCS}_{p}$ signifies the contribution of source p to heavy metal I; $\left|{b}_{pi}\times \overline{{APCS }_{p}}\right|$ signifies the absolute average contribution; and ${RC}_{pi}$ signifies the contribution rate of source p to heavy metal i.

## Results

### Heavy metal content in Beilun District in 2015 and 2022

The concentration of heavy metals at the 60 sampling sites in Beilun District are shown in Table 2. According to Table 2, the order of heavy metal content from high to low over the two years was Zn > Pb > Cr > Cu > Ni > As > Cd > Hg. Except for Ni and Hg in 2015, the average concentrations of heavy metals in Ningbo were higher than the background values of the soil, but they were all within the GB15618-2018 and GB36600-2018. Table 2 shows that the coefficients of variation (CV) of Cr, Cu, Zn, Cd and Pb in 2015 were 165.29%, 463.68%, 91.82%, 90.85% and 428.91%, respectively, and the CVs of Hg, Cu and Cd in 2022 were 322.08%, 101.68% and 93.20%, respectively. It shown that the contents of these heavy metals are highly dispersed and influenced by human activities. From the comparison of the medians, it can be seen that except for Zn, the heavy metal content in 2022 was higher than that in 2015, which indicates that heavy metals accumulated during these years.

**Table 2:** Heavy metal content in Beilun District in 2015 and 2022.

Year	Heavy metals	Minimum (mg/kg)	Maximum (mg/kg)	Average (mg/kg)	Median (mg/kg)	Geometric mean (mg/kg)	Standard deviation	Coefficient of variance (%)	Soil background value* (mg/kg)
2022	Hg	0.05	7.33	0.31	0.12	0.13	1.00	322.08	0.255
	Cr	16.12	222.91	68.89	63.63	57.69	40.59	58.93	62.1
	Ni	6.23	49.69	26.51	24.87	23.09	12.69	47.87	28.8
	Cu	7.61	200.04	32.51	23.68	24.80	33.05	101.68	21.1
	Zn	49.61	404.70	124.95	90.44	106.02	86.21	69.00	99.8
	As	2.14	33.87	15.60	14.12	13.93	7.02	45.00	7.2
	Cd	0.05	1.05	0.25	0.16	0.19	0.24	93.20	0.123
	Pb	28.12	157.65	53.74	43.16	49.06	27.68	51.51	28.1
2015	Hg	0.02	0.32	0.09	0.08	0.08	0.06	64.51	0.255
	Cr	12.72	1143.00	88.73	62.20	62.38	146.67	165.29	62.1
	Ni	2.86	157.00	27.26	20.48	21.44	22.73	83.37	28.8
	Cu	2.45	3068.00	85.27	22.16	23.07	395.38	463.68	21.1
	Zn	44.18	930.30	169.02	124.40	136.64	155.18	91.82	99.8
	As	2.80	28.86	9.74	8.93	9.06	4.06	41.69	7.2
	Cd	0.03	0.91	0.22	0.16	0.17	0.20	90.85	0.123
	Pb	20.50	3318.00	98.76	35.44	42.09	423.60	428.91	28.1

*The data is the background value of heavy metals in the soil of Ningbo, which is derived from The Soil Element Background Values in China, 1990.

### Heavy metal content in different land-use types

**Figure 2:**
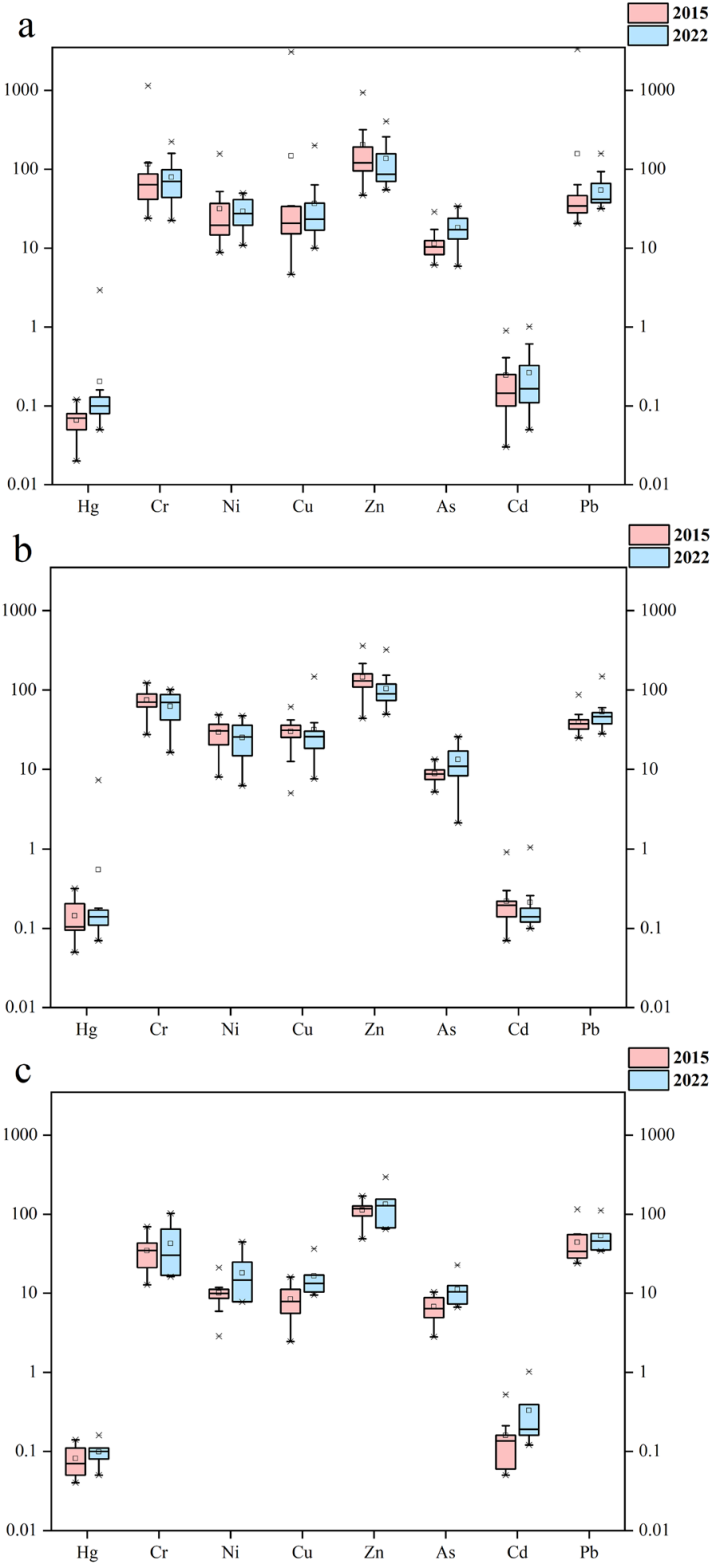
Heavy metal content in green belts (**a**), agricultural land (**b**), and woodland (**c**).

**Figure 3:**
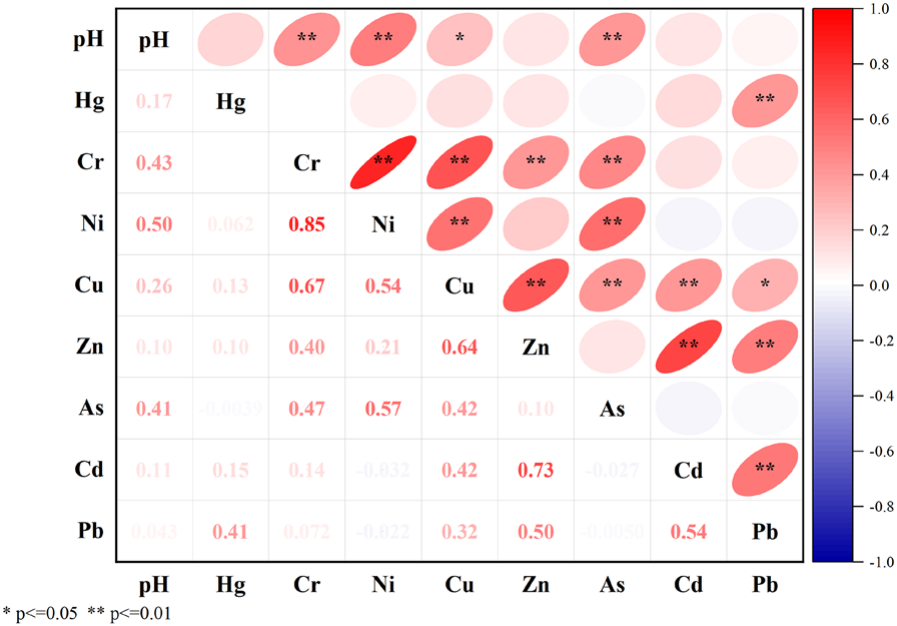
Correlation of heavy metal content (*p ≤ 0.05 indicate a significant correlation, while **p ≤ 0.01 indicate a highly significant correlation).

The heavy metal contents corresponding to various land use types in 2015 and 2022 are shown in Fig. 2. The heavy metal contents of green belts and woodland in 2022 were greater than those in 2015, and there were no significant changes in the heavy metal content of agricultural land. The Hg content decreased in the order agricultural land > green belts > woodland. The Zn content in 2022 decreased in the order green belts > woodland > agricultural land, the Cd content decreased in the order woodland > green belts > agricultural land, and the concentration of the remaining heavy metals decreased in the order green belts > agricultural land > woodland. This trend is also reflected in the pH results. The Pearson correlation coefficients between heavy metals and pH in Beilun District are shown in Fig. 3. pH was significantly correlated with Cr, Ni, Cu and As. Significant correlations were also observed for Hg with Pb; Cr with Ni, Cu, Zn, and As; Cu with Cd, Pb; and Zn with Cd and Pb.

### Comparison of spatiotemporal distribution of heavy metals

**Figure 4:**
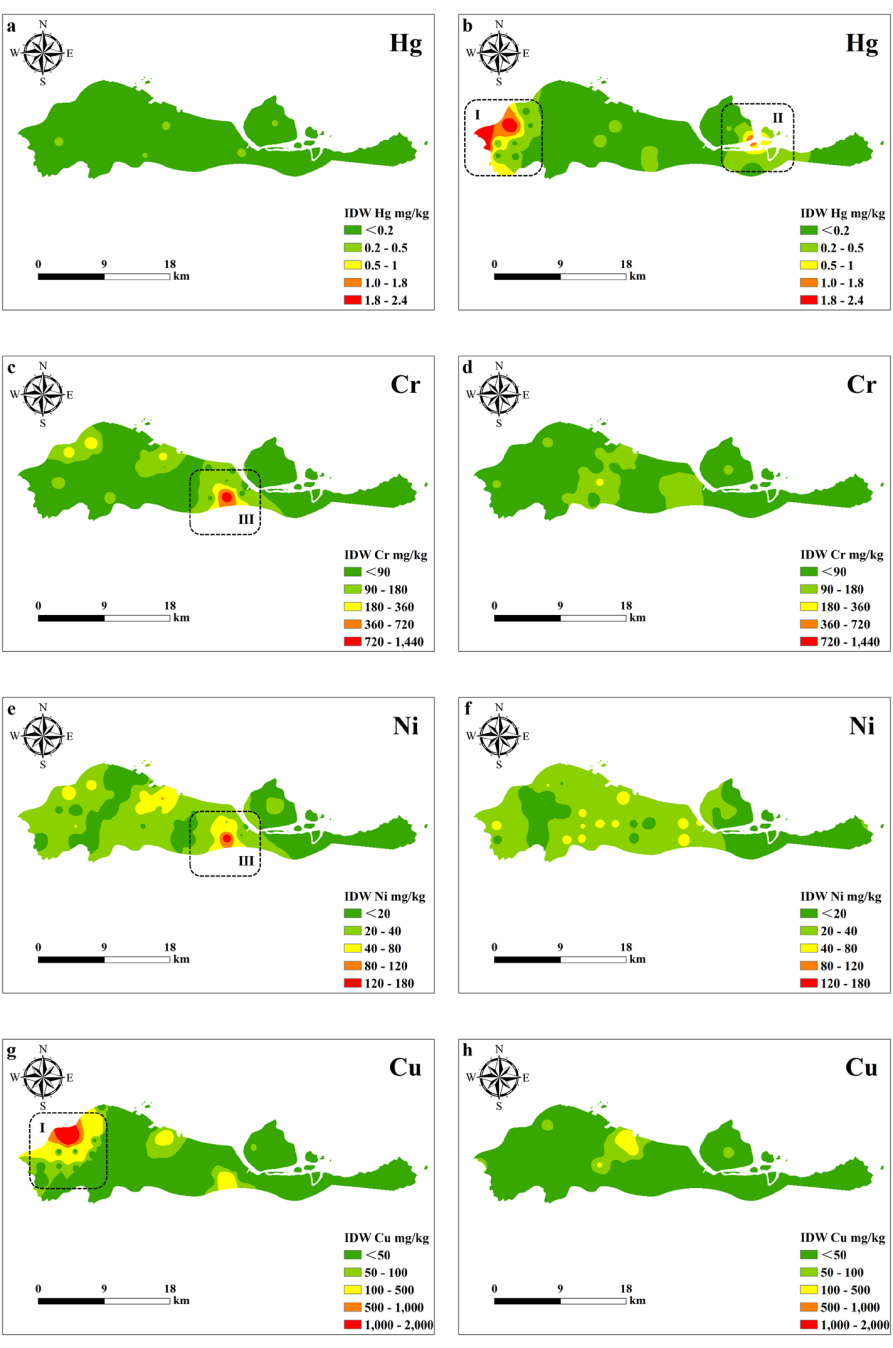

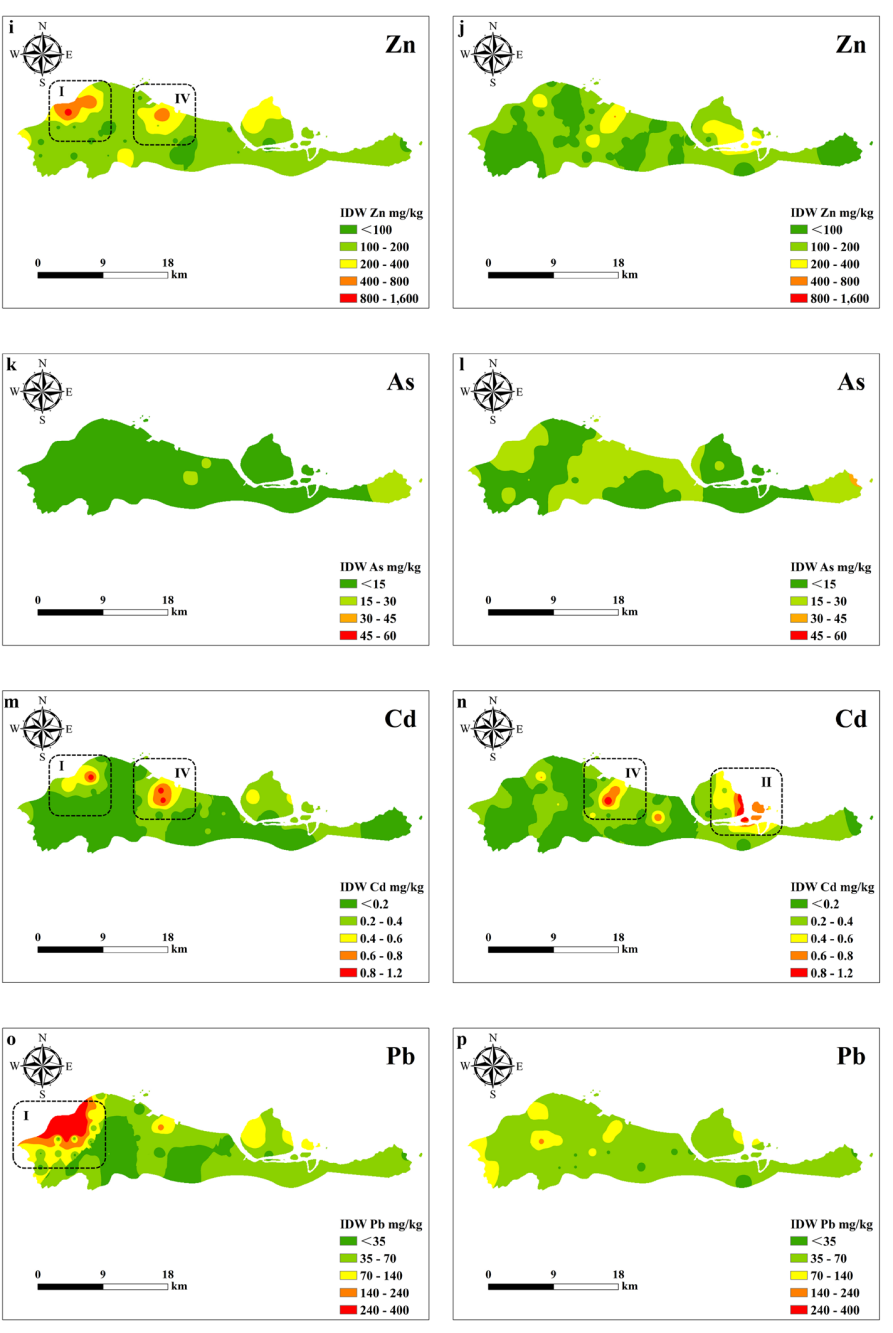
Spatial–temporal distribution of heavy metals in 2015 (**a,c,e,g,i,k,m,o**) and 2022 (**b,d,f,h,j,l,n,p**) (I: Western, Xiaogang; II: Northeast, Daxie; III: Northwestern, Suanshan; IV: Central, Chaiqiao).

The spatiotemporal distribution of heavy metals in Beilun District in 2015 and 2022 is shown in Fig. 4. As shown in Fig. 4, the heavy metal content in Beilun District varies little spatially, and the areas with higher heavy metal contents are mainly concentrated in the western, northwestern, northeast and central regions. These four regions correspond to the Xiaogang Hufang Industrial Zone, the Ningda Industrial Zone, Xinqiao Chemical Co., the Wanhua Industrial Park, the Formosa Plastics Industrial Park, and the Huang’an Industrial Zone. This indicates that the sources of the high heavy metal contents in Beilun District may be these industrial areas as well as various factories and chemical enterprises. The distribution of heavy metal contents varied over time. In 2015, the concentrations of Cu, Zn, Cd and Pb were higher in the northwest, and the content of Cr was higher in the central region. In 2022, the contents of Hg and Cd were higher in the northeast, the Ni content was higher in the central region, and the Hg content was also higher in the west.

### Heavy metal pollution degree analysis

**Figure 5:**
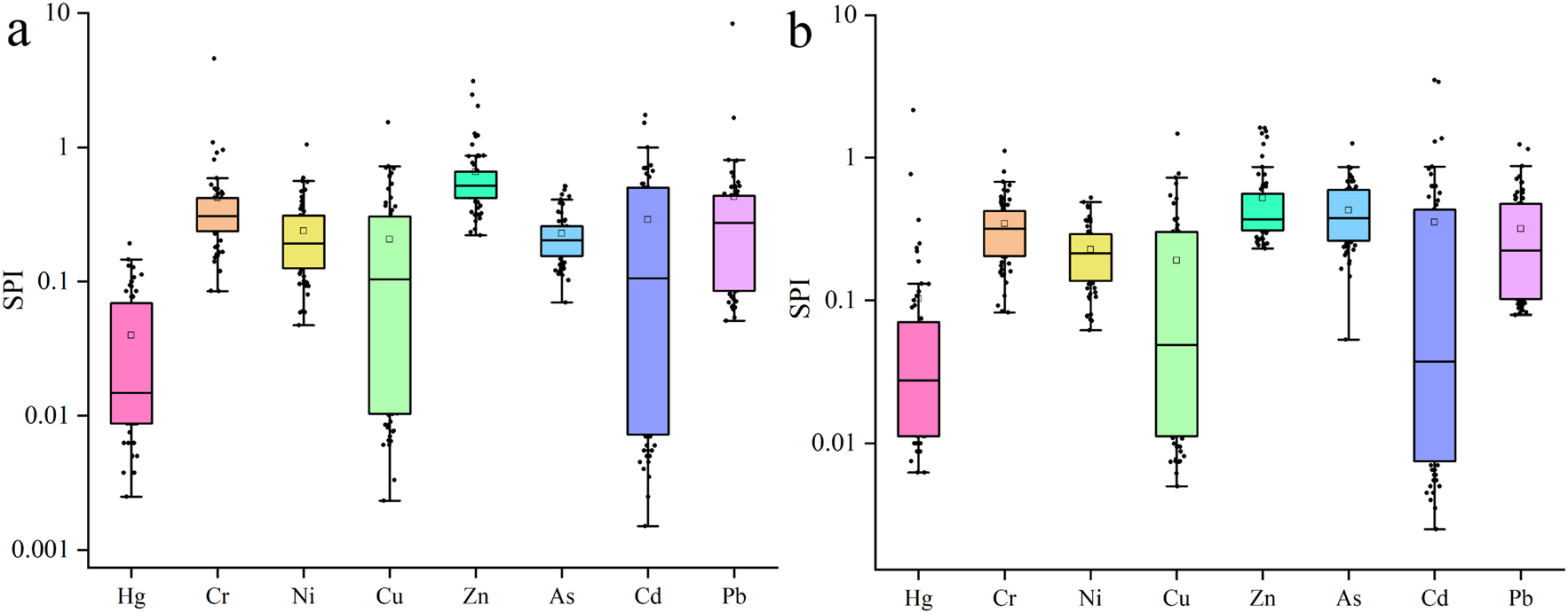
Evaluation of single-factor pollution index in 2015 (**A**) and 2022 (**B**).

The single-factor pollution index, PLI, and I_geo_ were used to analyze the heavy metal content to reflect the comprehensive heavy metal pollution in Beilun District. The single-factor pollution index results are shown in Fig. 5. Most of the heavy metals have a single-factor index of less than 1, which indicates that their content is within the standard range. There are also a few points with a single-factor index of higher than 1, and the maximum value of the single-factor index in 2015 is higher than that in 2022. This indicates that heavy metals exceed the standard at a few points in Beilun District, and the pollution level in 2015 was greater than that in 2022.

Table 3 shows the PLI results, which intuitively show the difference in pollution status in different regions. The PLI of woodland areas indicates no pollution, and those of green belts and agricultural land indicate a moderate load. This indicates that human activities greatly affect the accumulation of heavy metals in green belts and agricultural land. The heavy metal content in 2022 increased compared with that in 2015; this accumulation was substantial in woodland areas and green belts, while there was no significant change in agricultural land.

**Table 3:** Pollution load index evaluation results.

Type of land use	Year	Quantity	PLI	PLI level
Woodland	2015	10	0.6202	Clean
	2022	7	0.9026	Clean
Green belts	2015	30	1.0718	Moderately load
	2022	32	1.1993	Moderately load
Agricultural land	2015	20	1.1017	Moderately load
	2022	21	1.0923	Moderately load

I_geo_ can account for the background differences caused by different pollution sources. As shown in Table 4, except for As, Cd, and Pb in 2022, which had levels of unpolluted to moderately polluted, the remaining heavy metals had unpolluted levels. This indicates that As, Cd, and Pb were the elements most strongly impacted by human activities. From the perspective of time, Hg, Ni, Cu, As, Cd, and Pb all accumulated from 2015 to 2022, and I_geo_ also increased.

**Table 4:** The results of geo-accumulation index evaluation.

Heavy metal	Year	Average	Maximum	Minimum	Median	Pollution grades
Hg	2015	− 2.25	− 0.26	− 4.26	− 2.26	Unpolluted
	2022	− 1.53	4.26	− 2.94	− 1.74	Unpolluted
Cr	2015	− 0.58	3.62	− 2.87	− 0.58	Unpolluted
	2022	− 0.69	1.26	− 2.53	− 0.55	Unpolluted
Ni	2015	− 1.01	1.86	− 3.92	− 1.08	Unpolluted
	2022	− 0.90	0.20	− 2.79	− 0.80	Unpolluted
Cu	2015	− 0.46	6.60	− 3.69	− 0.52	Unpolluted
	2022	− 0.35	2.66	− 2.06	− 0.42	Unpolluted
Zn	2015	− 0.13	2.64	− 1.76	− 0.27	Unpolluted
	2022	− 0.50	1.43	− 1.59	− 0.73	Unpolluted
As	2015	− 0.25	1.42	− 1.95	− 0.27	Unpolluted
	2022	0.37	1.65	− 2.34	0.39	Unpolluted to moderately polluted
Cd	2015	− 0.14	2.30	− 2.62	− 0.21	Unpolluted
	2022	0.05	2.51	− 1.88	− 0.21	Unpolluted to moderately polluted
Pb	2015	0.00	6.30	− 1.04	− 0.25	Unpolluted
	2022	0.22	1.90	− 0.58	0.03	Unpolluted to moderately polluted

According to the results of the three evaluations, there was no heavy metal pollution in Beilun District as a whole. Only a few points exhibited slight pollution or an excessive content of individual heavy metals.

### Pollution source analysis

**Figure 6:**
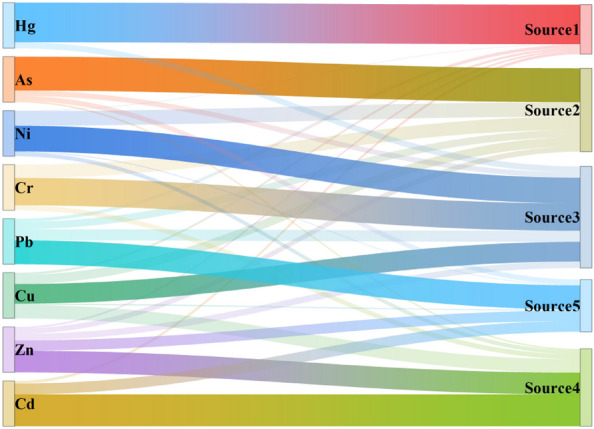
PMF heavy metal contribution.

The contribution rates of 5 sources to 8 heavy metals were determined by PMF analysis, as shown in Fig. 6. Hg was most affected by source 1, with a contribution rate of 86.90%, which was much higher than the rates for other heavy metals. As was most influenced by source 2, with a contribution rate of 74.20%. Cr, Ni and Cu were greatly affected by source 3, with contribution rates of 60.30%, 56.50% and 43.10%, respectively. Zn and Cd were more influenced by Source 4, contributing 47.60% and 70.40%, respectively. This source also contributed 29.50% to Cu, indicating that Cu was affected by both source 3 and source 4. Pb was most influenced by source 5, contributing 52.80%, while this source contributed 22.80% and 23.60% to Zn and Cd, respectively.

After reducing the heavy metal dimensionality, three factors were obtained. The cumulative variance contribution was 79.80%, and the total variances were 34.07%, 30.23% and 15.50%. The PCA results are shown in Table 5. The first factor accounted for 34.07%; the main contributing elements were Cr, Ni, Cu and As, and the loading coefficients were 0.89, 0.93, 0.67 and 0.75, respectively. The second factor accounted for 30.23%; the main contributing elements were Cu, Zn, Cd and Pb, and the loading coefficients were 0.58, 0.90, 0.90 and 0.64, respectively. The third factor accounted for 15.50%; the main contributing elements were Hg and Pb, and the loading coefficients were 0.95 and 0.56, respectively.

**Table 5:** Principal component score.

Heavy metal	Factor1	Factor2	Factor3
Hg	0.04	0.047	**0.953**
Cr	**0.887**	0.229	− 0.063
Ni	**0.931**	− 0.008	0.029
Cu	**0.666**	**0.575**	0.061
Zn	0.234	**0.9**	0.007
As	**0.752**	− 0.069	0.031
Cd	− 0.056	**0.899**	0.081
Pb	− 0.051	**0.64**	**0.561**
Eigenvalue	2.726	2.418	1.24
Variance interpretation rate%	34.074	30.228	15.497
Cumulative variance explanation rate%	34.074	64.302	79.799

Significant values are in bold.

**Figure 7:**
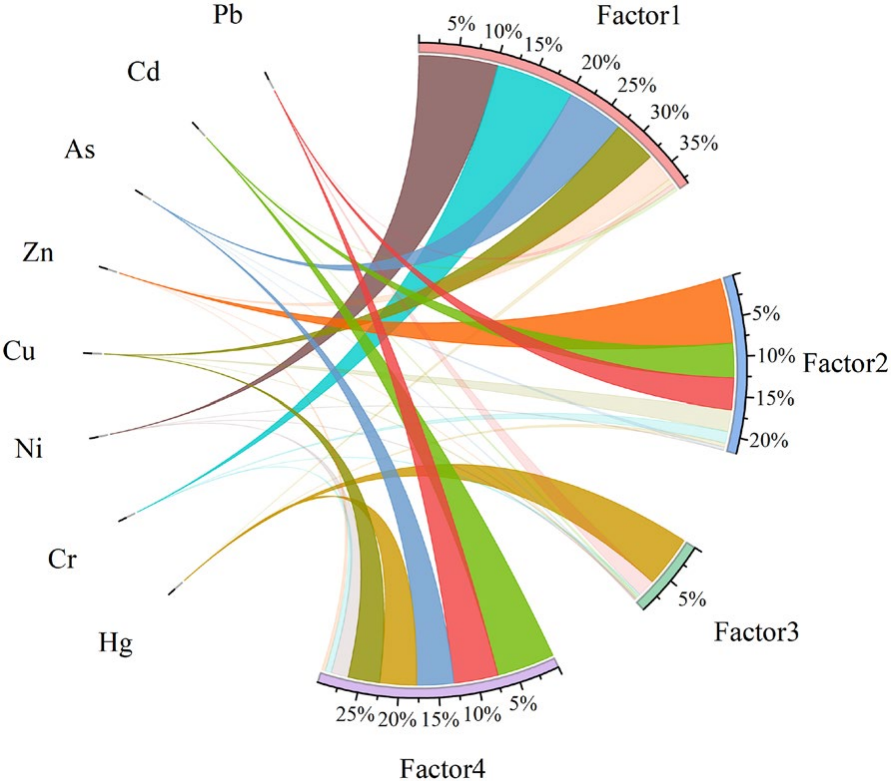
PCA-APCS-MLR heavy metal contribution.

The contribution rate of each heavy metal is shown in Fig. 7. Cr, Ni, Cu and As were the most affected by the first factor, with contribution rates of 79.55%, 80.95%, 44.04% and 58.10%, respectively. The second factor had the highest contribution rates to Zn, Cd and Pb, which were 66.35%, 35.22% and 32.16%, respectively. The third factor had the highest contribution rate to Hg, which was 52.56%, much higher than those of other heavy metals. The fourth factor was calculated from the constant term of MLR and is generally interpreted to indicate other anthropogenic sources. The heavy metals with high contribution rates were Hg, Cu, As, Cd and Pb, with contribution rates of 37.52%, 32.25%, 37.87%, 59.66% and 45.42%, respectively.

## Discussion

### Distribution of heavy metals

The heavy metal content of various land use types in Beilun decreased in the order green belts > agricultural land > woodland, and the PLI showed the same trend. This impact may be attributed to human activities, such as industry and transportation, which have caused the accumulation of heavy metals in green belts. The accumulation degree of heavy metals was lower in agricultural land than in green belts. This is because agricultural land and woodland at the most marginal levels of urban and peri-urban areas are least affected by human activities and therefore have the lowest levels of heavy metal accumulation^24,50^. Huang and Shi et al. also found that the heavy metal content of various land use types decreased in the order mining areas > industrial areas > agricultural land > woodland^28,29^.

Compared with that in 2015, the Zn content in 2022 decreased slightly, and there was no significant change in Cd, while the contents of other heavy metals increased. From 2015 to 2022, the Zn content in the soil in Beilun District decreased slightly, there was no significant change in Cd content, and the contents of other heavy metals increased. This indicates that heavy metals such as Hg, Cr, Ni, Cu, As, and Pb accumulated over the 7 years. According to the spatiotemporal distribution results, eight heavy metals were detected in the five industrial zones of Beilun, corresponding to point source pollution. There were some similarities in the spatial distributions of Cr and Ni, the distributions of Cu, Zn and Cr, and the distributions of Pb and Hg. Pathak et al. also found that Cu, Zn, Ni and Cr have the same spatial distribution^51^. Over time, point source pollution of Cr, Ni, As, Zn, Pb, and Cu disappeared or decreased. Differences or similarities in the temporal variation in heavy metals are associated with the geochemical characteristics of the elements, human activities and sources^52^. In recent years, the Chinese government has implemented several measures to control pollution sources. For instance, in 2016, they released the Soil Pollution Prevention and Control Action Plan^53^. The Ningbo Municipal Government has also actively responded to the call of the state and has made achievements in reducing emissions and controlling pollution. However, such pollution prevention measures do not restrict the use of pesticides and herbicides in agricultural land, so the As content in Beilun increased over the 7 studied years^34^. From 2015 to 2022, the point source pollution of Hg increased, and the Cd pollution area moved. These changes may be related to atmospheric deposition from coal combustion in thermal power plants and nearby industrial activities^54,55^. We should pay attention to this phenomenon.

### Heavy metals pollution source analysis

According to the PMF analysis, there are five heavy metal sources in Beilun District, and source one contributes the most to Hg. The average content of Hg in Beilun District exceeded the soil background value in Ningbo by 1.22 times, and the CV was 322%. This indicates that the human factors greatly affected the Hg content. Wang et al. showed that Hg pollution in the environment mainly comes from the smelting of nonferrous metals and coal-fired thermal power plants, while Hg-containing catalysts are often used in the oil cracking process and finally enter the environment via industrial “three waste” emissions^56–58^. Considering the spatial distribution of Hg observed in this work, Hg pollution is mainly concentrated in the western and northeastern regions, which contain Tianyi Petrochemical Co., the Beilun Power Plant, and various hardware manufacturing and chemical enterprises, such as Xiebei Thermal Power Co. and Daxie Petrochemical Co. Therefore, it is inferred that source 1 is a chemical source.

As is most influenced by source 2. The As content in Beilun District is 2.17 times greater than the background value in Ningbo. This high value indicates severe accumulation of As. From the spatial distribution and land-use type analysis, it was found that most of the areas with high As contents are located in farmland. This indicates that As pollution in Beilun District is mainly farmland pollution. Zhang et al. showed that the use of pesticides, insecticides and herbicides causes As accumulation, and Wang et al. further showed that the soil As content with multiple pesticide applications is 200 times that in the absence of pesticides^59,60^. Therefore, source 2 is inferred to be an agricultural source.

Cr, Ni and Cu are greatly affected by source 3. The average contents of Cr and Ni are close to the background values in Ningbo’s soil, and the CV is low. This indicates that human activities have a lesser impact on Cr and Ni. The average content of Cu is slightly higher than the background value, and the CV is large, indicating a strong influence of human activities. This indicates that Cu was also affected by other sources in addition to source 3. Therefore, it is inferred that source 3 is a natural source. Xia et al. showed that the contents of Ni and Cr are mainly related to the parent material of the soil, and Li et al. and Shen et al. reached similar conclusions^61–63^.

Zn and Cd are more influenced by source 4. Cu is also affected by source 4, indicating that Cu is affected by both source 3 and source 4. Zn, Cd, and Cu are often considered to be characteristic of metal smelting and industrial electroplating activities, and they are also related to automobile exhaust emissions and tire wear. According to the spatial distribution results, Cd, Zn, and Cu pollution is mainly concentrated in the north and northeast. These two areas include many machinery, auto parts, and electronics manufacturing plants; therefore, it is inferred that source 4 is related to electroplating and machinery manufacturing. Wu et al. and Wu et al. confirmed this view^16,64^.

Pb is most influenced by source 5. Pb, Zn, and Cd are the hallmark pollutants of traffic pollution. Studies have shown that incomplete combustion of automobile fuel produces Pb pollution, tire friction with the ground produces Zn pollution, and the loss of automobile parts produces Cd pollution^39,65,66^. Therefore, it is inferred that source 5 is a traffic source.

The APCS-MLR and PMF analyses yielded similar source results. On the basis of the PMF analysis and spatial distribution, it can be seen that the first factor is natural and agricultural sources, the second factor is traffic sources, the third factor is chemical sources, and the fourth factor is other anthropogenic sources^67^. Considering the CV and I_geo_ values, the fourth factor may be a combination of industrial and agricultural sources.

**Figure 8:**
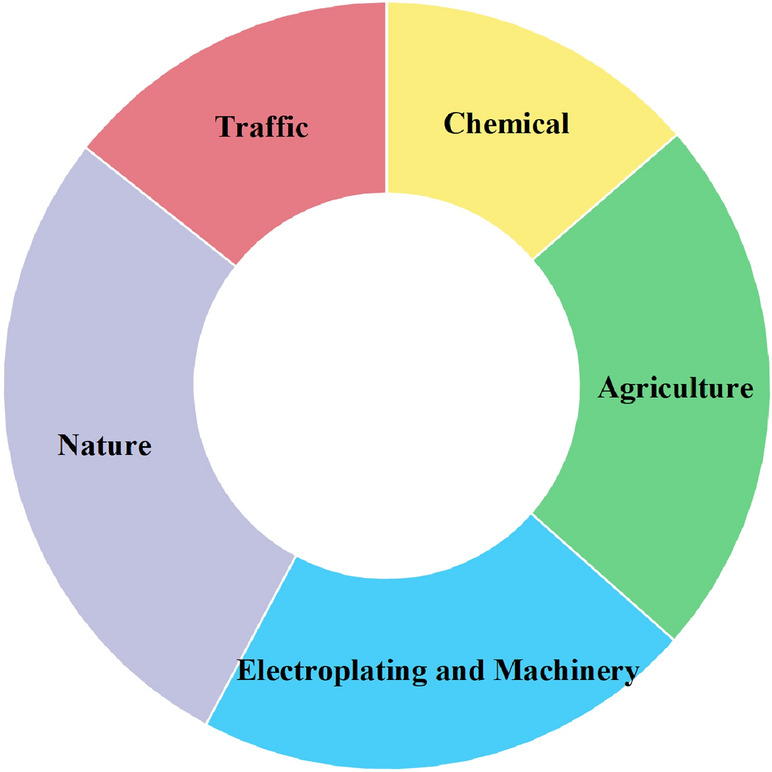
Source of heavy metals.

In summary, a total of five pollution sources were identified by combining the results of PMF and APCS-MLR analysis. The results, corresponding to natural sources, agricultural sources, chemical sources, electroplating and machinery manufacturing sources and traffic sources, are shown in Fig. 8.

## Conclusion

The spatial–temporal distribution, land type differences, pollution degree and source of eight heavy metals in Beilun District of Ningbo were studied. Heavy metals in Beilun District are predominantly concentrated in five industrial parks. Over time, compared to 2015, there has been an accumulation in heavy metal content by 2022. However, there has been a significant decrease in points with extremely high heavy metal content in 2022, which may be related to environmental protection policies in Ningbo City. The heavy metal contents of various land use types decreased in the order green belts > agricultural land > woodland. According to the results of the three pollution evaluations, there was no heavy metal pollution in Beilun District as a whole, and only some heavy metals at individual points were slightly polluted. PMF and APCS-MLR analysis revealed five pollution sources: Hg-based chemical sources, As-based agricultural sources, Cr, Ni, and Cu-based natural sources, Zn and Cd electroplating and machinery manufacturing sources, and Pb-based traffic sources. The results of this study offer a reference for future research on heavy metals in urban soil, and the source analysis results provide a reference and guidance for subsequent soil heavy metal pollution control.

## Data Availability

We affirm that all data are authentic and unaltered and are freely available for academic research and verification by any researcher. If there are any inquiries about the data, please contact the lead author or submit queries via the following email address: yyli@iue.ac.cn.
